# Supplementary material for: Development of an artificial intelligence-enhanced warfarin interaction checker platform
Source: PLOS Digit Health. 2025 Mar 24;4(3):e0000756. doi: 10.1371/journal.pdig.0000756 (PMC11932461; doi:10.1371/journal.pdig.0000756)
Supplement: Attachment — Submitted filename: Response to Reviewers.docx [file pdig.0000756.s002.docx]

**Dear editor of PLOS Digital Health,**
We sincerely thank you and the reviewer for the insightful comments and suggestions regarding our manuscript titled “*Development of an Artificial Intelligence- Enhanced platform Warfarin interaction Checker: Using Python Programming Language to Facilitate Patients Access and Minimize Medication Errors*" (Submission ID: PDIG-D-24-00492) submitted to PLOS Digital Health. We have carefully addressed each comment and made the corresponding revisions to improve the quality of our work. Below, we provide a point-by-point response to each comment.

**Reviewer #1:**

**Comment 1:**

*“Title and Formatting: Please review the manuscript title for grammatical consistency, particularly regarding hyphenation and capitalization. Consider revising to "Development of an Artificial Intelligence-Enhanced Warfarin Interaction Checker Platform."*

**Response:**

Thank you for this valuable suggestion. We agree that the proposed title better reflect the scope and the focused of our manuscript. Accordingly, we have revised the title to the suggested title by the reviewer to “**Development *of an Artificial Intelligence-Enhanced Warfarin Interaction Checker Platform****”*

**Comment 2:**

*“Methods and Results: Include brief quantitative metrics from your beta testing phase to support the platform's effectiveness. A concise summary of user satisfaction scores or system accuracy measurements would be valuable. Please add a short description of the validation methodology used to verify the system's accuracy in identifying drug interactions.”*

**Response:**

Thank you for your suggestion to provide a summary of user satisfaction scores. To address this, we distributed an anonymous survey to both pharmacists and patients who tested the application. The survey assessed their satisfaction across five domains: **Website Performance, Usability and Satisfaction, Feature Effectiveness, Visual and Information Clarity, and Overall Impact**. We have now included a summary of the survey results in the manuscript. Additionally, we invited respondents to leave comments.

**Validation Methodology:**

To ensure the accuracy of the system in identifying drug interactions, beta testing was conducted with healthcare professionals and patients. This involved real-world testing scenarios to assess interaction detection accuracy, usability, and reliability. Continuous feedback was incorporated iteratively to refine the platform.

The detailed summary of these findings has been added to the **Method** and **Results section** of the manuscript. We hope this addition improves the evaluation of the application.

**Comment 2:**

*“Technical Implementation: Provide a brief overview of the security measures implemented for handling medical information. This addition will address an important consideration for clinical implementation. If your goal is to demonstrate technical feasibility of the approach only, then this would not be needed, but if so, include a comment as to have this may be achieved in a clinical implementation.”*

**Response:**

Thank you for raising the point regarding security measures for handling medical information. In this project, the Warfa-Check platform was developed primarily as a simple demonstration to show how artificial intelligence can identify warfarin interactions with drugs, food, and herbs. The platform does not handle or store any personally identifiable information or sensitive medical records. Users interact with the application anonymously, and all queries are processed in real time without retaining any data. Since the tool is designed to avoid data storage or exchange, no specific security measures for safeguarding medical information are required at this stage. We provided also a brief overview regarding this point in the manuscript and added it to the method section. We hope this overview clarifies the point made regarding the technical implication.

We sincerely appreciate your valuable comments and feedback. We hope the revised manuscript meets your expectations and standards.
